# Effects of phantom exercises on pain, mobility, and quality of life among lower limb amputees; a randomized controlled trial

**DOI:** 10.1186/s12883-021-02441-z

**Published:** 2021-10-27

**Authors:** Anna Zaheer, Arshad Nawaz Malik, Tahir Masood, Sahar Fatima

**Affiliations:** 1 Faculty of Rehabilitation and Allied Health Sciences, Riphah International University, Lahore, Pakistan; 2 Faculty of Rehabilitation and Allied Health Sciences, Riphah International University, Islamabad Campus, Rawalpindi, Pakistan; 3 Department of Physical Therapy and Health Rehabilitation, College of Applied Medical Sciences, Jouf University, Sakakah, Saudi Arabia; 4 Faculty of Allied Health Sciences, University Institute of Physical Therapy, The University of Lahore, Lahore, Pakistan

**Keywords:** Mirror therapy, Mobility, Phantom limb pain, Phantom exercises. Quality of life

## Abstract

**Background:**

The objective of the current study is to evaluate the effects of phantom exercises on phantom limb pain, mobility status, and quality of life in lower limb amputees treated with mirror therapy and routine physiotherapy.

**Methods:**

It is a randomized controlled trial in which 24 unilateral lower limb amputees (above and below the knee) were randomly assigned to two equal groups i.e., control group (mirror therapy and conventional physical therapy) and experimental group in which, phantom exercises were given, additionally. Physical therapy included conventional therapeutic exercises while phantom exercises include imagining the movement of the phantom limb and attempting to execute these movements Data were collected at baseline, after 2 and 4 weeks of intervention using VAS (pain), AMP (mobility) and RAND SF-36 Version 1.0 (QOL) questionnaires. All statistical analyses were done with IBM SPSS 25.0 with 95% CI.

**Results:**

Twenty-four amputees (17 males and 7 females) participated in this trial. The Mean age of the participants in experimental and control groups was 45.3 ± 11.1 years and 40.5 ± 12.5 years respectively. After the intervention, the pain (VAS score) was significantly lower in the experimental group (*p* = 0.003). Similarly, the experimental group demonstrated a significantly better score in the “bodily pain” domain of SF-36 (*p* = 0.012). Both groups significantly (*p* < 0.05) improved in other domains of SF-36 and ambulatory potential with no significant (*p* > 0.05) between-group differences.

**Conclusions:**

The Addition of phantom exercises resulted in significantly better pain management in lower limb amputees treated with mirror therapy and routine physiotherapy.

**Trial registration:**

This study is registered in the U.S National Library of Medicine. The clinical trials registration number for this study is NCT04285138 (ClinicalTrials.gov Identifier) (Date: 26/02/2020).

**Supplementary Information:**

The online version contains supplementary material available at 10.1186/s12883-021-02441-z.

## Introduction

Limb amputation negatively affects the psychological, social and physical health of the patients [1]. Phantom limb pain (PLP) is a common complaint after lower limb amputation can be defined as discomfort or pain in a missing part of the limb. According to the literature, the incidence of PLP ranges from 42.2 to 78.8% of all cases, while the reported prevalence is 45–85% [2]. Although PLP subsides with time in most patients irrespective of the cause of amputation, it persists for several years in 5–10% of cases [3]. PLP is distinguishable from residual limb pain (RLP) since RLP originates from physical impairments such as skin conditions, vascular abnormalities, the impaired healing process, neuromas, soft tissue and bone disorders, etc. [4, 5].

One of the foremost causes of PLP is a history of chronic pain (e.g., due to diabetes or peripheral vascular disease) in the affected limb before amputation. Other causative factors include seasonal changes, infections, defecation movements, urination, etc. [6]. Multiple mechanisms are reported to be involved in PLP, such as spinal, supraspinal and peripheral although there is no consensus on the exact mechanism of PLP [7–9] and it is difficult to treat condition for amputees, therefore management of PLP should be mechanism-based and multimodal in nature, while keeping in mind the spinal, peripheral and supra-spinal methods rather than conventional therapeutic techniques [10].

More than two dozen strategies can be found in the literature for the management of PLP. However, there is no broad consensus on the best and most effective option. One of the rehabilitation strategies for PLP which has shown promise in recent years is mirror therapy (MT). In MT, a mirror is placed in a way that allows an amputee to view the reflection of the sound limb which is positioned near the mirror and its reflection is visualized by the patient while the residual limb is placed behind the mirror. This position creates an illusion of having both extremities intact, afterwards the patient moves intact extremity in different patterns. The Exact mechanisms of action for MT remains uncertain. However, reintegration of sensory and motor systems, control over avoiding fear and restoration of body image might play a role. Nevertheless, it is inexpensive, safe and easy to administer therapy [11]. One of the limitations of this therapy is the unclear reliability of visual feedback of amputated limbs [12].

One of the less investigated strategies for the management of PLP is phantom motor execution (PME), also known as phantom exercises. PME involves the imaginary movement of phantom limb in the brain along with the performance of certain actual physical movements. Neurophysiological networking involved in PME is similar to that of actual executed physical activities of sound limb and it should be distinguished from pure imaginary activities as it follows a different neurophysiological pathway [13]. Such exercises have been shown to safely and effectively relieve PLP in various types of limb amputations [14]. For instance, the effectiveness of phantom exercises – versus general exercises - was evaluated in post-traumatic lower limb amputees. A Significantly greater reduction in pain was observed as a result of phantom exercises [15]. in which the mirror image is replaced by a computer-generated graphical representation of the lost limb.

As an alternative to MT, virtual reality training (VRT) can also be used to manage PLP, especially in patients with bilateral limb amputations. In VRT computerized graphical representation of amputated limb is used instead of traditional mirror. Currently, a number of medical-related programs using virtual reality have been established and used in clinical practice for the rehabilitation of various diseases. In a study conducted on “resonance behaviours” in monkeys, it was observed when monkeys actually perform a behaviour or only observe a behaviour, premotor cortex is activated on a similar level, therefore, it is suggested that mirror neurons are the neurological background in VRT. These techniques using virtual reality have obvious benefits in that it makes available feasible control of the environmental factors as compared to other therapeutic techniques [16]. Despite the effectiveness of immersive virtual reality training, its uses are limited due to the high cost. PLP may deteriorate several important concepts of health-related quality of life (QOL) such as mood, sleep, independence, emotional health or relationship with family and friends, etc. [17] This suggests us to find novelty in strategies to prevent, reverse or manage this painful condition specially in case if it becomes chronic and interfere with patient’s quality of life [14].

Phantom limb pain among lower limb pain is highly prevalent in Pakistan [18]. Moreover, the effects of phantom exercises, in conjunction with mirror therapy, for managing PLP in lower limb amputees are largely unknown and according to researcher’s knowledge no study has been conducted to assess effectiveness of phantom exercises among amputees in Pakistan. Because of the high prevalence of PLP and its consequences on the physical and mental health of amputees, there is a need to design an easily administered, home-based and effective treatment protocol. Therefore, the main objective of the current study was to evaluate the effects of phantom exercises on phantom limb pain, mobility status, and quality of life in lower limb amputees treated with mirror therapy and routine physiotherapy. It was hypothesized that the addition of phantom exercises would result in significantly improved clinical outcomes in all studied parameters.

## Methods

### Study design

The current single-blind (participants) randomized controlled trial was conducted in accordance with the guidelines outlined in the declaration of Helsinki. The ethical approval for the study was obtained from the ethics committee of the Riphah International University, Lahore, Pakistan. All patients provided written informed consent before participation. This study is registered in the U.S National Library of Medicine. The clinical trials registration number for this study is NCT04285138 (ClinicalTrials.gov Identifier) (Date: 26/02/2020).

### Setting

Pakistan Society for the Rehabilitation of the Disabled (PSRD) and Hope foundation for rehabilitation sciences, Lahore, Pakistan.

### Patient information

**Fig. 1:**
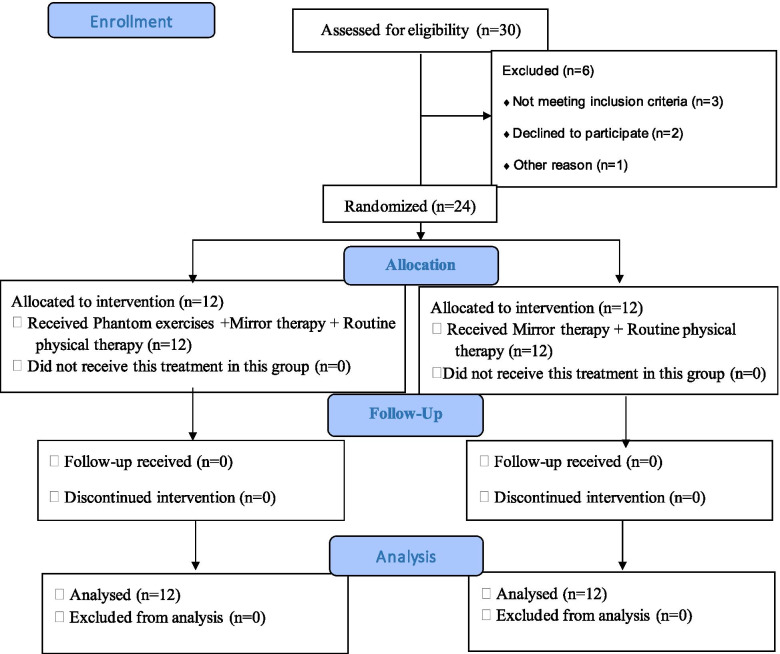
CONSORT flow diagram of the trial

The Sample size is 6 in each group calculated by openEpi [19]. We included 12 patients in each group. The patients were recruited via non-probability convenience sampling. Thirty lower limb amputees were screened for eligibility through Limb Deficiency and Phantom Limb Questionnaire among other criteria. Twenty-four eligible patients were randomly assigned to two equal groups by sealed envelope method: experimental or control (Fig. 1). Both male and female amputees – aged 18 to 60 years - with phantom limb pain (≥4 on VAS) were eligible for this trial. Unilateral lower limb amputees (above and below the knee) were included while those with psychological/neurological impairments, visual impairments, infectious stump/residual limb, hearing impairment were excluded from the study.

### Rehabilitation protocols

Both study groups received mirror therapy (15 min) and routine physical therapy (20 min) while the experimental group was given Phantom Motor Execution (PME), also known as phantom exercises, additionally (15 min).

### Phantom motor execution

Phantom exercises included the imagining movement of the phantom limb and attempting to execute these movements. The patients were asked:

1. About the position at which they were feeling their phantom limb.
2. To place their intact limb at the same position as they felt their phantom limb.
3. To move their both limbs in opposite direction.
4. To return to their starting position. The movements included ankle inversion/eversion, flexion/extension, adduction with flexion of toes as clenching, abduction with the extension of toes as unclenching. After the patient feels relaxed, movements as knee flexion/ extension or hip flexion/ extension were repeated, until the PLP disappeared [19]. Phantom exercises were repeated until the PLP subsided completely with a maximum of 15 repetitions in one session.

### Mirror therapy

A standing flat mirror (130 cm × 46 cm) with a wooden frame, base (62 cm × 65 cm) was used to perform 15 min of mirror therapy under the physiotherapist’s supervision every day for 4 weeks. Mirror was placed parasagittal near patient’s body in such a way reflective surface is towards the sound limb. Amputees were enabled to see reflection of their sound limb in that mirror. Rules of mirror therapy were instructed and patient was instructed that his/her eyes should be always focused on reflection of intact limb in the mirror and symmetrical movements should be performed. Patient can freely decide which movements he/she wants to repeat in front of the mirror. Mirror therapy was practiced daily. It should be emphasized that consistency in therapy is significant to achieve expected outcomes. Sources of external stimulations (noise, television) were minimized, all accessories on the sound limb were removed, and the patient’s comfort was ensured before the start of each session. The goals and benefits of mirror therapy were explained to the patients who were encouraged to ask questions.

The reflection of their sound limb mimics the amputated limb, and movement of intact limb gives illusion as if amputated limb is moving without any pain. Mirror therapy is believed to influence cortical reorganization as it exploits the brain’s referencing the visual information over somatosensory feedback. Therefore, it can reverse maladaptive cortical reorganization causing a reduction in phantom limb pain. MT is also seen to be increasing cortical and spinal motor excitability.

### Routine physiotherapy

The routine physical therapy programme was consisted of stretching of tight muscles, strengthening, isometric, dynamic, mobilizations, prosthesis/gait training according to the level of amputation and their assessment outcomes. Subjects in the control group were advised to continue their rehabilitation at physiotherapy departments as frequent as possible. Participants were also advised to keep a log of the nature, frequency and duration of their physical activities [15, 20].

### Measurement tools

Limb Deficiency and phantom limb questionnaire was used for evaluating the limb amputees in detail at the time of recruitment [21]. Additionally, Amputee Mobility Predictor (AMP) was used, which is an easy and reliable way of assessing the ambulatory status of lower limb amputees, with or without the prosthesis. AMP consists of 21 different tasks with a maximum overall score of 39 signifying best ambulatory potentials. High inter- and intra-rater reliability have been reported for AMP in the literature. The severity of phantom limb pain was subjectively assessed with the Visual Analogue Scale (VAS) [22]. Quality of life was evaluated through RAND 36-Item Health Survey (Version 1.0). It is a valid and reliable tool to assess the quality of life among both diseased and healthy adults (either gender) [23, 24]. Although few kinds of research used SF-36 total scoring, it has been discouraged by both the SF-36 scoring manual as well as developers [25]. SF-36 scores can also be converted into two different components including the Physical mental component (GH, PF, BP and RP) and Mental component summary (SF, VT, RE and MH) but in this study, eight health domains were addressed as recommended by RAND scoring manual: physical functioning, bodily pain, role limitations due to physical health problems, role limitations due to personal or emotional problems, emotional well-being, social functioning, energy/fatigue, and general health perceptions [26]. Scores included in each domain was converted in scale i.e., worst (0) to best (100).

### Data collection

Twenty-four patients were randomly allocated into two groups; Interventional group and control groups. To ensure that the patients knew the differences between residual limb, phantom limb, and between phantom limb pains and postoperative wound pains rather than a questionnaire, a proper interview was conducted. Demographics and detail information about their amputation was collected using Limb Deficiency and phantom Limb Questionnaire. After which participants were guided about their treatment plan and therapist demonstrated all relevant exercises. A specially designed Boucher having illustrations and detailed information about mirror therapy and phantom exercises was also provided to all participants. For the convivence of responders in both groups all the instruction given verbally or written in brochure. The brochure was written in native language Urdu. After first assessment and treatment amputees were asked to continue their exercise plan at home and they were reassessed after every week. During home-based treatments, daily telephonic reminders were given to all of the participants. Research evidence recommends that a treatment protocol of 4 weeks of mirror therapy may reduce chronic pain. So, the treatment lasted for 4 weeks and data was collected at baseline and 2-week intervals until the conclusion of 4-week interventions by using VAS, AMP and SF-36 questionnaires. All assessments and random allocation of participants were performed by the same physical therapist (AZ) at all stages of data collection for all patients.

### Data analysis

The Normality of the data was assessed through Shapiro–Wilk test. Repeated measures ANOVA was used to analyse training-induced changes in both groups. Between-group differences were computed with independent samples t-test. All statistical analyses were done with IBM SPSS 25.0 (IBM, New York, USA). Alpha level of significance was set at 0.05 with a confidence interval of 95%.

## Results

Out of 24 participants, the number of male patients in the experimental and control groups was 8 and 9 respectively. The mean (standard deviation) age of patients in the experimental and control group was 45.3(11.1) years and 40.5(12.5) years correspondingly. The Vast majority of the patients underwent amputation within 2 years before this trial (experimental group =10; control group = 11). Most patients reported right-handedness except for 1 patient in each group, 9(37.5%) were comorbid along with amputation. 10(41.67%) participants underwent amputation due to diabetic neuropathy, 12(50%) underwent amputation due to any traumatic event. The Majority of the patients were married. Both groups were comparable at baseline with regard to all variables. Table 1 represents between group differences in all variables measured through independent sample t-test.

**Table 1:** Between-group change scores for phantom limb pain (VAS score), quality of life domains (SF-36 scores) and mobility status (Amputee mobility predictor score) through independent t test

Variables		Experimental group Mean ± SD	Control group Mean ± SD	***P***-Value
Visual Analogue Scale	**Week 2**	3.33 ± 0.89	4.08 ± 1.45	0.182
	**Week 4**	2.25 ± 0.621	3.58 ± 1.24	**0.003**
Physical functioning	**Week 2**	51.0 ± 11.1	53.9 ± 4.7	0.409
	**Week 4**	56.7 ± 9.4	56.3 ± 6.8	0.902
Physical role limitations	**Week 2**	56.7 ± 13.5	56.7 ± 6.6	0.130
	**Week 4**	60.0 ± 13.0	61.0 ± 7.6	0.292
Bodily pain	**Week 2**	56.4 ± 15.5	53.7 ± 13.2	0.644
	**Week 4**	72.9 ± 16.2	55.4 ± 15.0	**0.012**
General health perceptions	**Week 2**	62.1 ± 16.3	53.7 ± 6.9	0.514
	**Week 4**	64.56 ± 13.4	61.8 ± 7.1	0.536
Energy/vitality	**Week 2**	56.7 ± 7.2	58.3 ± 11.9	0.682
	**Week 4**	61.3 ± 8.3	62.5 ± 14.1	0.793
Social functioning	**Week 2**	53.7 ± 14.5	57.5 ± 12.3	0.502
	**Week 4**	55.4 ± 14.2	59.6 ± 12.3	0.451
Emotional role limitations	**Week 2**	56.7 ± 17.2	56.3 ± 16.4	0.952
	**Week 4**	58.3 ± 17.2	56.7 ± 16.8	0.813
Mental health	**Week 2**	58.8 ± 11.3	57.6 ± 13.4	0.807
	**Week 4**	61.3 ± 12.1	58.3 ± 13.2	0.578
Amputee Mobility predictor	**Week 2**	21.25 ± 2.96	21.50 ± 2.96	0.887
	**Week 4**	25.92 ± 4.12	24.33 ± 4.61	0.385

### Pain

According to longitudinal comparison within groups, the pain was significantly (*p* = 0.000, F value = 15.453) improved in both experimental (4.4167 ± 1.50 vs. 3.34 ± 0.9 vs. 2.25 ± 0.621) and control group (4.33 ± 1.67 vs.4.08 ± 1.45 vs. 3.58 ± 1.24) after 2 and 4 weeks of treatment. But both groups remained comparable after 2 weeks as no difference was seen between both groups ((*p* = 0.182) at this stage. However, the experimental group proved to be significantly (*P* = 0.003) better in decreasing pain as compared to the control group after 4 weeks of training.

### Quality of life

Participants included in this study showed significant (*p* < 0.05) improvement in all domains of quality of life in SF-36, as measured through within group comparisons. F values of physical functioning, physical role limitations, bodily pain, general health perceptions, energy/vitality, social functioning, emotional role limitations and mental health domains are 19.315, 24.485, 15.061, 9.634, 16.357, 13.513, 1.279 and 5.509, respectively. Both groups were statistically similar concerning all domains except “bodily pain” after 4 weeks, where the experimental group reported a better score than the control group (72.9 ± 16.2 vs. 55.4 ± 15.0, *P* = 0.012).

### Ambulatory potential

Neither group improved significantly in the first 2 weeks but there was significant (*p* = 0.000, F value = 28.695) training-induced improvement in both groups i.e.; experimental (20.83 ± 3.64 vs 25.92 ± 4.12) and control group (21.08 ± 5.45vs 24.33 ± 4.61) after 4 weeks. Both groups were comparable at all stages of data collection. However, there is no significant (*p* = 0.385) difference between both groups in improving the mobility status of amputees. Only the experimental group exceeded the minimal detectable change of 3.4 points, on average.

## Discussion

The present study aimed to evaluate the effects of phantom exercises on phantom limb pain, mobility status, and quality of life in lower limb amputees treated with mirror therapy and routine physiotherapy for 4 weeks. Although it was hypothesized that the addition of phantom exercises will lead to improvement in all parameters, the findings support a significantly beneficial effect of additional phantom exercises on pain only. This benefit was evident after 4 weeks of training.

Neurophysiological mechanisms involved in phantom motor execution (phantom exercises) are very similar to actual executed physical activities. These movements are hypothesized to have two effects at cortical level i.e., one is that while executing phantom execution movements motor area corresponds to absent limb and second it improves motor control of residual musculature in stump enlarge its representation in cortex into absent limb area as those neural resources are less used due to amputation [27]. The results of current study support earlier finding by Raffin and co-workers who reported that a reduction of phantom movements leads to higher severity of phantom limb pain [13]. Hence, better control over phantom movements, accomplished through phantom exercises, could explain a greater reduction in pain severity in the experimental group.

Additionally, it should be noted that no significant change occurred in either study group in the first 2 weeks of training followed by significant improvement in both groups, much more so in the phantom exercisers. This delayed response may suggest a late control achieved by the patients over the phantom movements as training progressed. It was termed as paralyzed phantom when patients were unable to execute movements in missing limbs initially but gained control over their phantom lower limbs after sufficient repetitions eventually [28].

Following amputation, the area that represents the lost limb in Primary Somatosensory Cortex do not receive its primary input, consequently that somatosensory body map boundaries changes. This process is called reorganisation. Current study suggests that the PE in combination with mirror therapy and conventional physical therapy exercises protocol can reverse this process. This shows when phantom exercises are used in combination of different mental imaginary exercises, it actually tricks patient’s brain as if their painful phantom limb is painless and can move easily thus reducing their phantom limb pain. These findings are consistent with the results of a randomized controlled trial conducted by Brunelli et al. in which a combination of progressive muscle relaxation, mental imagery, and modified phantom exercises was recommended as an effective regimen to relieve pain and other sensations related to a phantom limb [27]. Similar results are seen in a study conducted to compare the effects of general exercises with and without phantom exercises, this study reported significantly reduced PLP in the phantom exercise group [15].

Quality of life among lower limb amputees are mostly compromised due to their disability [18]. Both, the perception of body image and PLP leads to variations in psychological profile, physical functional outcomes and quality of life scores of amputees [4]. According to results of current study, participants in both groups showed improvements in all domains of quality of life. These changes can be attributed to regular physical therapy including stretching, strengthening, static, prosthetic/gait training and dynamic exercises which may facilitate wound healing and metabolism [29]. Third domain of SF-36 representing bodily pain status showed maximum improvement out of all domains throughout the treatment. More improvement was seen in interventional group because phantom exercises in combination of mirror therapy seems to be valuable strategy in reducing phantom limb pain, possibly due to its effect on activation of pre-motor cortex [19].

In terms of ambulatory potential, neither study group demonstrated a significant change in the initial 2 weeks of training. However, statistically significant improvement was seen in both groups during the remaining 2 weeks. The ambulatory status was comparable between the groups indicating no additional benefit of phantom exercises. The improved ambulatory status can be explained by the physiotherapy regimen which was administered to both groups [30]. Hence, these findings suggests that phantom exercises are not superior over MT or conventional physical therapy treatments in improving mobility status and quality of life domain (except bodily pain domain) among lower limb amputees. Nevertheless, it should be noted that only the experimental group exhibited the minimal detectable change of 3.4 points which was proposed earlier [31]. This signifies that although statistically, both regimes were comparable, the addition of phantom exercises leads to better clinical outcomes.

Studies conducted on chronic stroke patients showed that sensory stimulation combined with the robot assisted virtual reality and mirror therapy has positive effects on upper limb functions [16, 32]. So virtual reality [33] seems to be an effective treatment approach that tricks the brain assuming that missing limb is still intact, properly functioning and pain free, eliminating phantom limb pain but its cost is an unsolved issue in developing countries such as Pakistan. In addition, mirror therapy has also proved to be a promising rehabilitation procedure that cause reintegration of sensory and motor systems, control over avoiding fear and restoration of body image which reduces phantom pain but evidence regarding efficacy of mirror box therapy is not definitive. Therefore, in this study combination of inexpensive, safe and easy to administer therapies such as PE, mirror therapy and routine physical therapy were used [34].

The main limitation of our study is that rehabilitation centres were closed because of the pandemic situation, which caused to difficulty in data collection and small sample size, which lead to the difficulty in generalizing the study findings. Another limitation is that location and cause of lower limb amputation were not taken into account while calculating results. Similarly, the absence of a record for the medication history might have influenced the findings. Myoelectric study of the residual limb may also increase the objectivity of the results. In future studies, subgroups based on demographics on larger sample size should be analysed with combinations of different treatments to find precise, home-based, low-cost treatment protocols for amputees suffering phantom limb pain. Moreover, the difference between motor imaginary and motor execution should also be taken into account while designing treatment strategies.

## Conclusion

The Addition of phantom exercises resulted in significantly better pain management in lower limb amputees treated with mirror therapy and routine physiotherapy for 4 weeks. However, no added effect of these exercises was seen on the ambulatory potential and most domains of the quality of life.

## Supplementary Information

**Additional file 1.**

## Data Availability

The datasets generated and analysed in the current study are available from the corresponding author on reasonable request.

## Abbreviations

PLP: Phantom limb pain
RLP: Residual limb pain
LLA: Lower limb amputation
QOL: Quality of life
AMPRO: Amputee Mobility predictor
MT: Mirror Therapy
VAS: Visual Analog Scale
VR: Virtual reality
PE: Phantom exercises
RCT: Randomized Controlled Trial
SF-36: 36-Item Short-Form Health Survey questionnaire
