# Supplementary material for: Effects of phantom exercises on pain, mobility, and quality of life among lower limb amputees; a randomized controlled trial
Source: BMC Neurol. 2021 Oct 27;21:416. doi: 10.1186/s12883-021-02441-z (PMC8554869; doi:10.1186/s12883-021-02441-z)
Supplement: Supplementary file 1 — Additional file 1. [file 12883_2021_2441_MOESM1_ESM.docx]

**EFFECTS OF PHANTOM EXERCISES ON PAIN, MOBILITY, AND QUALITY OF LIFE AMONG LOWER LIMB AMPUTEES; A RANDOMIZED CONTROLLED TRIAL**

ClinicalTrials.gov identifier (NCT number): NCT04285138. (Date: 26/02/2020)

**Anna Zaheer**

Student, Faculty of Rehabilitation and Allied Health Sciences. Riphah International University, Lahore, Pakistan (annazaheer@yahoo.com)

**Arshad Nawaz Malik**

Professor, Faculty of Rehabilitation and Allied Health Sciences. Riphah International University, Islamabad Campus. (arshad.nawaz@riphah.edu.pk)

**Tahir Masood**

Associate Professor, Department of Physical Therapy and Health Rehabilitation, College of Applied Medical Sciences, Jouf University, Saudi Arabia. (tmasood@ju.edu.sa)

**Sahar Fatima**

Lecturer, University Institute of Physical Therapy, Faculty of Allied Health Sciences, The University of Lahore, Lahore, Pakistan. ([sehar.fatima@uipt.uol.edu.pk](mailto:sehar.fatima@uipt.uol.edu.pk))

- Study was conducted in Pakistan Society for the Rehabilitation of the Disabled (PSRD) and Hope foundation for rehabilitation sciences, Lahore, Pakistan.

**1. Project summary**

The objective of current study is to evaluate the effects of phantom exercises on phantom limb pain, mobility status, and quality of life in lower limb amputees treated with mirror therapy and routine physiotherapy. Twenty-four individuals with lower leg amputation participated in this trial and were randomly assigned to two equal groups. Limb Deficiency and Phantom Limb Questionnaire was used for screening of the participants. Other tools were Visual analogue scale (pain), Amputee mobility predictor (Ambulatory status) and 36-Item Short-Form Health Survey questionnaire (Health-related quality of life). The study was conducted in 12 months and data obtained was analysed through Statistical Package for Social Sciences (SPSS) 25.0 (*IBM, New York, USA*). The intervention lasted for 4 weeks. The control group underwent mirror therapy and conventional physical therapy while the experimental group performed phantom exercises additionally. Data was collected at baseline, after two and four weeks of intervention using. Mean age of the participants in experimental and control groups was 45.3±11.1 years and 40.5±12.5 years respectively. Both groups were comparable at baseline. After intervention, pain was significantly lower in the experimental group (p<0.05). Similarly, the experimental group demonstrated significantly better score in the “bodily pain” domain of SF-36 (p<0.05). Both groups improved in other domains of SF-36 and ambulatory potential with no significantly between-group differences after intervention. It was concluded that addition of phantom exercises resulted in significantly better pain management in lower limb amputees treated with mirror therapy and routine physiotherapy.

**2. Rationale & background information**

Limb amputation negatively affects psychological, social and physical health of the patients (1). Phantom limb pain (PLP) is a common complaint after lower limb amputation can be defined as discomfort or pain in missing part of the limb. According to the literature, the incidence of PLP ranges from 42.2% to 78.8% of all cases, while the reported prevalence is 45-85% (2). Although PLP subsides with time in most patients irrespective of the cause of amputation, it persists for several years in 5-10% cases (3). PLP is distinguishable from residual limb pain (RLP) since RLP originates from physical impairments such as skin conditions, vascular abnormalities, impaired healing process, neuromas, soft tissue and bone disorders, etc. (4, 5).

One of the foremost causes of PLP is history of chronic pain (e.g., due to diabetes or peripheral vascular disease) in the affected limb before amputation. Other causative factors include seasonal changes, infections, defecation movements, urination, etc. (6). Multiple mechanisms are reported to be involved in PLP, such as spinal, supraspinal and peripheral although there is no consensus on the exact mechanism of PLP (7, 8). It has been suggested that multiple neurological mechanisms are responsible for PLP and RLP (9). PLP may deteriorate several important concepts of health-related quality of life (QOL) such as mood, sleep, independence, emotional health or relationship with family and friends, etc. (10)

One of the methods for subjectively evaluating the limb amputees in detail is limb Deficiency and phantom limb questionnaire (11). Similarly, several strategies and resources are available for evaluating the mental and physical health of amputees during rehabilitation. One of such tools is Amputee Mobility Predictor (AMP) which is an easy and reliable way of assessing ambulatory status of lower limb amputees, with or without the prosthesis. The severity of phantom limb pain can be subjectively assessed with Visual Analogue Scale (12). As for quality of life, one of the more commonly used instrument is 36-Item Short Form Survey (SF-36) which evaluates eight health domains: physical functioning, bodily pain, role limitations due to physical health problems, role limitations due to personal or emotional problems, emotional well-being, social functioning, energy/fatigue, and general health perceptions (13).

More than two dozen strategies can be found in the literature for the management of PLP. However, there is no broad consensus on the best and most effective option. One of the rehabilitation strategies for PLP which has shown promise in recent years is mirror therapy (MT). In MT, a mirror is placed in a way that allows amputee to view the reflection of the sound limb which is positioned near the mirror and its reflection is visualized by the patient while the residual limb is placed behind the mirror. This position creates an illusion of having both extremities intact, afterwards the patient moves intact extremity in different patterns. This tricks patient’s brain as if their painful phantom limb is painless and can move easily. Exact mechanisms of action for MT still remains uncertain. However, reintegration of sensory and motor systems, control over avoiding fear and restoration of body image might play a role. Nevertheless, it is inexpensive, safe and easy to administer therapy (14). One of the limitations of this therapy is the unclear reliability of visual feedback of amputated limb. (15)

One of the less investigated strategy for the management of PLP is phantom motor execution (PME), also known as phantom exercises. PME involves the imaginary movement of phantom limb in the brain along with performance of certain actual physical movements. Neurophysiological networking involved in PME is similar to that of actual executed physical activities of sound limb and it should be distinguished from pure imaginary activities as it follows a different neurophysiological pathway (16). Such exercises have been shown to safely and effectively relieve PLP in various types of limb amputations (17). For instance, the effectiveness of phantom exercises – versus general exercises - was evaluated in post-traumatic lower limb amputees. Significantly greater reduction in pain was observed as a result of phantom exercises (18).

As an alternative to MT, virtual reality training (VRT) can also be used to manage PLP, especially in patients with bilateral limb amputations. Despite the effectiveness of immersive virtual reality training, it uses are limited due to high cost. Because of high prevalence of PLP and its consequences on physical and mental health of amputees, there is a need to design easily administered, home-based and effective treatment protocol. The effects of phantom exercises, in conjunction with mirror therapy, for managing PLP in lower limb amputees are largely unknown. Therefore, the main objective of the current study was to evaluate the effects of phantom exercises on phantom limb pain, mobility status, and quality of life in lower limb amputees treated with mirror therapy and routine physiotherapy. It was hypothesized that addition of phantom exercises would result in significantly improved clinical outcome in all studied parameters.

**3. Study design**

The current single-blind randomized controlled trial was conducted in accordance with the guidelines outlined in the declaration of Helsinki.

**4. Methodology**

*4.1 Patient information*: The mean (standard deviation) age of patients in the experimental and control group was 45.3(11.1) years and 40.5(12.5) years correspondingly. Vast majority of the patients underwent amputation within 2 years prior to this trial (experimental group =10; control group = 11). The number of male patients in experimental and control group was 8 and 9 respectively. Most patients reported right-handedness except for 1 patient in each group. The patients were recruited via non-probability convenience sampling. Thirty lower limb amputees were screened for eligibility through Limb Deficiency and Phantom Limb Questionnaire among other criteria.

*4.2 Eligibility Criteria:*

*4.2.a Inclusion Criteria:*

- Ages Eligible for Study: 18 Years to 50 Years (Adult)
- Accepts Healthy Volunteers: No
- Unilateral lower limb amputation
- Phantom limb pain score (40-100 on VAS)
- Either gender
- Stable prosthetic situation (i.e., satisfaction with the fitting of the prosthesis) or being a non-user.

*4.2.b Exclusion Criteria:*

- Amputees with psychological/neurological impairments.
- Amputees having neuropathic pain other than phantom limb pain.
- Patients having visual-spatial impairments.
- Taking pain relief medications will also be excluded.
- Patients having residual limb pain.
- Inability to give informed consent.
- Carcinoma
- Infectious stump
- Severe hearing loss
- Any condition that restricts the movement of opposite limb, pain or limited range of motion in the intact limb
- Infectious and systematic diseases

*4.3 Sample size:* Sample size is 6 in each group calculated by openEpi (24). We included 12 patients in each group.

*4.4 Setting:* Pakistan Society for the Rehabilitation of the Disabled (PSRD) and Hope foundation for rehabilitation sciences, Lahore, Pakistan.

*4.5 Rehabilitation protocols*: Both study groups received mirror therapy (15 minutes) and routine physical therapy (20 minutes) while the experimental group was given Phantom Motor Execution (PME), also known as phantom exercises, additionally (15 minutes).

*4.5.a Phantom Motor Execution:* Phantom exercises included imagining movement of the phantom limb and attempting to execute these movements. The patients were asked:

1. About position at which they were feeling their phantom limb.
2. To place their intact limb at same position as they felt their phantom limb.
3. To move their both limbs in opposite direction.
4. To return back to their starting position. (24)

The movements included ankle inversion/eversion, flexion/extension, adduction with flexion of toes as clenching, abduction with extension of toes as unclenching. After patient feel relaxation, movements as knee flexion/ extension or hip flexion/ extension were repeated, until the PLP disappeared. (19) Phantom exercises were repeated until the PLP subsided completely with a maximum of 15 repetitions in one session.

*4.5.b Mirror therapy*: A standing flat mirror was used to perform 15 minutes of mirror therapy under physiotherapist’s supervision every day for 4 weeks. Sources of external stimulations (noise, television) were minimized, all accessories on the sound limb were removed, and patient’s comfort was ensured before the start of each session. Goals and benefits of mirror therapy were explained to the patients who were encouraged to ask questions. Pain level was measured on VAS prior to each session and rules of mirror therapy were instructed.

*4.5.c Routine physiotherapy*: The regimen comprised stretching of the tight musculature, static and dynamic muscle strengthening. Participants were also advised to keep a log of nature, frequency and duration of their physical activities. (20)

*4.6 Primary Outcome Measures:*

*4.6.a Visual analogue scale (VAS)* [ Time Frame: 4 weeks]

changes from the baseline, the visual analogue scale The VAS evaluates pain subjectively. It consists of a 100-mm line, with two endpoints representing "no pain" to "worst pain imaginable from left to right." Patients are instructed to mark on the line according to the level of pain and the same is measured

*4.6.b Amputee Mobility predictor* [ Time Frame: 4 weeks]

changes from the baseline, Amputee mobility predictor (AMP) is an amputee-specific tool for predicting ambulatory potential. This clinical test evaluates the predicted mobility of amputee with and without a prosthesis The AMP is a clinical test consisting of 20 tasks that are given a score of 0, 1, or 2 based on the amputee's performance. There is an item 21 where a score is given ranging from 0 to 5.

*4.6.c Limb Deficiency and Phantom Limb Questionnaire* [ Time Frame: day 1]

Patients will be evaluated using "Limb Deficiency and Phantom Limb Questionnaire (Questionnaire 2008, Version 2)" in order to assess their eligibility for including in this study.

This questionnaire does not have any scoring, it only concludes the questions on Yes/No basis for presence or absence of phantom limb pain.

*4.7 Secondary Outcome Measures:*

*4.7.a Short form 36 (SF-36)* [ Time Frame: 4 weeks]

changes from the baseline, the 36-Item Short-Form Health Survey questionnaire (SF-36) is a very popular instrument for evaluating Health-Related Quality of life (HRQOL). It is a widely used valid and reliable tool for measuring the (HRQOL) and it can be employed in both the healthy and diseased population such as amputees for assessing the outcome of health care services and the effect of medical intervention.

The SF-36 measures eight scales: physical functioning (PF), role physical (RP), bodily pain (BP), general health (GH), vitality (VT), social functioning (SF), role emotional (RE), and mental health (MH). Component analyses showed that there are two distinct concepts measured by the SF-36: a physical dimension, represented by the Physical Component Summary (PCS), and a mental dimension, represented by the Mental Component Summary (MCS).

*4.8 Equipment:*

Therapy mirror: A standing mirror (130 cm × 46 cm) with wooden frame and base (62 cm × 65 cm)

*4.9 Data collection*: Data was collected at baseline and at 2-week intervals until the conclusion of 4-week interventions. Visual Analogue Scale was used to quantify the intensity of pain before, during, and after the interventions. The ambulatory potential of the patients was assessed with the Amputee Mobility Predictor which consists of 21 different tasks with a maximum overall score of 39 signifying best ambulatory potential. High inter- and intra-rater reliability has been reported for AMP in the literature. Quality of life was evaluated through 36-Item Short Form Survey (SF-36) which addresses eight health domains: physical functioning, bodily pain, role limitations due to physical health problems, role limitations due to personal or emotional problems, emotional well-being, social functioning, energy/fatigue, and general health perceptions. 24 patients were randomly allocated into two groups; Interventional group and control groups. To ensure that the patients knew the differences between residual limb, phantom limb, and between phantom limb pains and postoperative wound pains rather than a questionnaire, a proper interview was conducted. Demographics and detail information about their amputation was collected using Limb Deficiency and phantom Limb Questionnaire. After which participants were guided about their treatment plan and therapist demonstrated all relevant exercises. A specially designed Boucher having illustrations and detailed information about mirror therapy and phantom exercises was also provided to all participants. For the convivence of responders in both groups all the instruction given verbally or written in brochure was in native language Urdu. All assessments were performed by the same physical therapist (AZ) at all stages of data collection for all patients.

*4.10 Data analysis*: Normality of the data was assessed through Shapiro–Wilk test. Multivariate analysis of variance was used to analyse training-induced changes in both groups. Between-group differences were computed with Mann-Whitney U test for VAS (pain) and second domain of SF-36. Independent samples t-test was used for AMP and remaining domains of SF-36. All statistical analyses were done with IBM SPSS 25.0 (*IBM, New York, USA*). Alpha level of significance was set at 0.05.

*4.11 Consort diagram:*


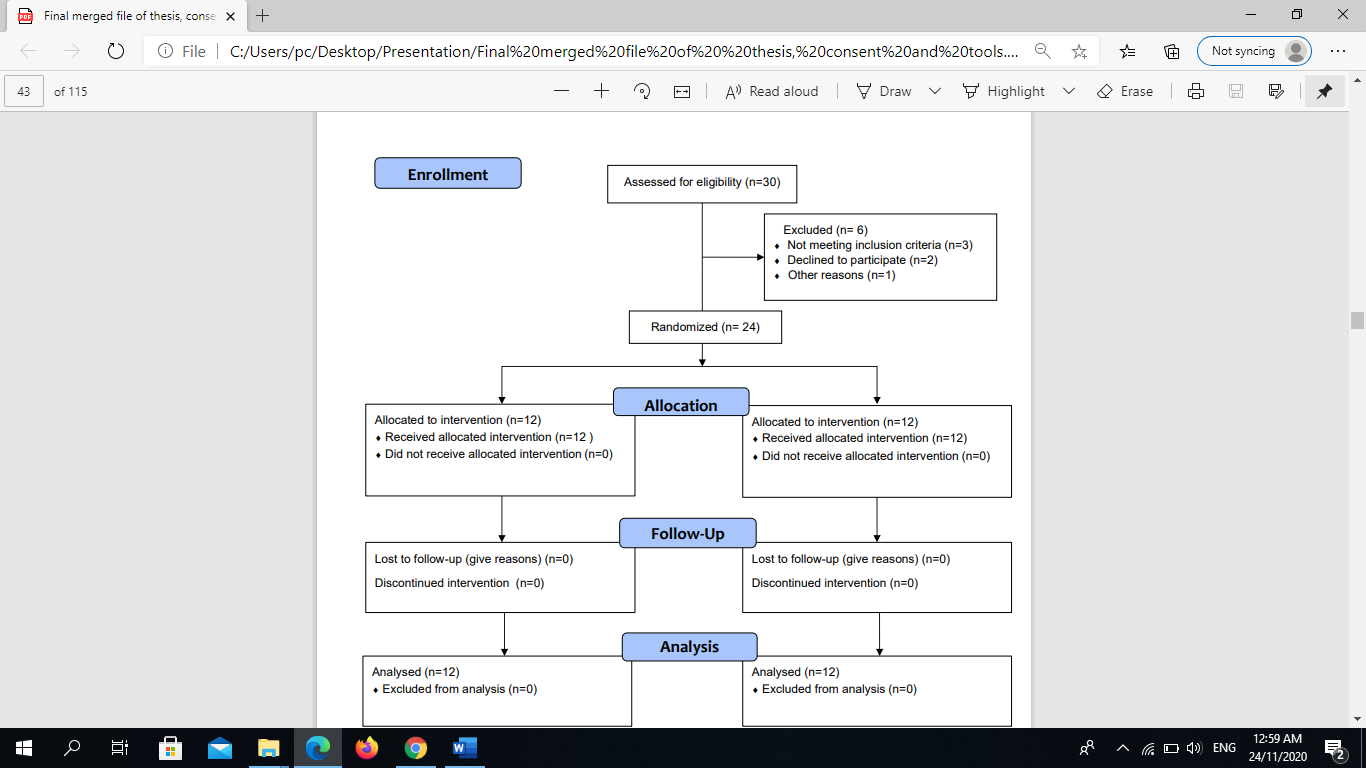


**5. Safety considerations**

- The participants were informed about the advantages of the study.
- Participants were ensured that there is no risk associated to treatment protocols given in this study
- They were informed that they are free to withdrawal their participation anytime during the study and they will not be penalized if they decided to leave.
- All ethical rules and regulations Ethical Committee of the University were followed while conducting the study while taking care of all rights of participants.
- All the participants were included in the study on the basis of written consent.
- Subjects were kept anonymous throughout the study and their identity will not be revealed in any of the publications.
- All information and collected data remained confidential.

**6. Follow-up**

After first assessment and treatment amputees were ask to continue their exercise plan at home and reassessed after two weeks. During home-based treatments, daily telephonic reminders were given to all of the participants. Research evidence recommends that a treatment protocol of four weeks of mirror therapy may reduce chronic pain. Therefore, therapist collected the data at baseline, and before and after treatment at the end of 2nd and 4th weeks.

**7. Data management and statistical analysis**

Normality of the data was assessed through Shapiro–Wilk test. Multivariate analysis of variance was used to analyse training-induced changes in both groups. Between-group differences were computed with Mann-Whitney U test for VAS (pain) and second domain of SF-36. Independent samples t-test was used for AMP and remaining domains of SF-36. All statistical analyses were done with IBM SPSS 25.0 (*IBM, New York, USA*). Alpha level of significance was set at 0.05.

**8. Quality assurance**

Yes, it was followed

**9. Expected outcomes of the study**

It was hypothesized that addition of phantom exercises would result in significantly improved clinical outcome in all studied parameters. Because of high prevalence and high pain intensity of phantom limb pain, there is need to find an effective, easily administered home based treatment for amputees, thus comparing effects of mirror therapy with and without phantom exercises in reducing pain and improving QOL and psychological status of amputees is a useful guide for future studies as these protocols are cost effective and efficient.

**10. Dissemination of results and publication policy**

It will be presented in conference, faculty meeting, professional gathering, publication. Etc

**11. Duration of the project**

Duration of project was 12 months after the approval of synopsis. (February 26, 2020 to March 30, 2021)

**12. Problems anticipated**

There may be lack of follow up or literacy ratio can affect outcome of study. Urdu based brochure helped natives to understand exercises and these problems were not faced during data collections.

**13. Project management**

AZ and SF were responsible for data control, monitoring intervention in order to detect any adverse effects, data analysis and manuscript writing. TM performed formal analysis, and validation of study data. ANM was responsible for the development of study design and project coordination as well as he reviewed and revised manuscript. All the authors further approved the final version of manuscript.

**15. Ethics**

Ethical approval was received from Ethical Review Committee of Riphah College of Rehabilitation and Allied Health Sciences, Riphah International University, Lahore (Reference number: REC/RCRS/20/2008). All ethical rules and regulations were followed while conducting the study and the participants were included in the study on the basis of informed consent.

**16. Informed consent forms**

Title of the Study: “Effects of Phantom exercises on pain, mobility and quality of life among lower limb amputees”

Principal Investigator Information:

Name: Anna Zaheer

Department: Faculty of Rehabilitation & Allied Health Sciences.

Email: annazaheer@yahoo.com

Phone: 0324-4765133

# *Purpose of study:*

• To compare the effects of Mirror therapy with and without Phantom exercise on phantom limb pain among lower limb amputees.

• To compare the effects of Mirror therapy with and without Phantom exercise on mobility status among lower limb amputees.

• To compare the effects of Mirror therapy with and without Phantom exercise on quality of life among lower limb amputees

## *Study Procedures:* This is a randomized control trial in which amputees having phantom limb pain will be randomly divided into two groups i.e., Experimental group (Routine physical therapy, mirror therapy and phantom exercises) and control group (Routine physical therapy and mirror therapy). Non- probability purposive sampling technique will be employed. Patients of age between 18 to 50 years and having phantom limb pain after lower limb amputation will be recruited by evaluating with limb deficiency and phantom limb questionnaire. Other tools will be Visual analogue scale (pain), Amputee mobility predictor (Ambulatory status) and 36-Item Short Form Health Survey questionnaire (Health-related quality of life). The study will be conducted in 6 months and data obtained will be analyzed.

*Risks:* None

*Benefits:* Since phantom limb remains painful several years after loss, there is need to find an effective, easily administered home-based treatment for amputees. Treatments use in this study can be beneficial in terms of reducing phantom limb pain among amputees. Moreover, this will be useful guide for future studies as these protocols are cost- effective and easy to perform.

*Confidentiality:* Your responses to this research work will be anonymous. Please do not write any identifying information on your survey. For the purposes of this research study, your comments will not be anonymous. Every effort will be made by the researcher to preserve your confidentiality.

*Compensation/Financial Consideration:* None

*Termination of research study:* You are free to choose whether or not to participate in this study. There will be no penalty or loss of benefits to which you are otherwise entitled if you choose not to participate. You will be provided with any significant new findings developed during the course of this study that may relate to or influence your willingness to continue participation. In addition, your participation in the study may be terminated by the investigator without your consent under certain circumstances.

*Contact Information in case of emergency:*

Dr. Anna Zaheer, PT Ph # 0324-4765133

*Voluntary participation:* Your participation in this research work is voluntary. It is up to you to decide whether or not to take part in this study. If you decide to take part in this study, you will be asked to sign a consent form. After you sign the consent form, you are still free to withdraw at any time and without giving a reason. Withdrawing from this study will not affect the relationship you have, if any, with the researcher. If you withdraw from the study before data collection is completed, your data will be returned to you or destroyed.

*CONSENT*

I have read and understand all the provided information and have had the opportunity to ask all relevant questions. I understand that my participation is voluntary and that I am free to withdraw at any time, without giving a reason and without any cost. I understand that I will be given a copy of this consent form. I voluntarily agree to take part in this research work.

Participant's Information:

Name:

Address:

Contact No.

Participant's signature Date

Investigator's signature Date

Signature of person obtaining consent Date

**17. References**

1. Solarz MK, Thoder JJ, Rehman S. Management of major traumatic upper extremity amputations. Orthopedic Clinics. 2016;47(1):127-36.

2. Ayoub SN, Hakim KY. Comparative study of dexmedetomidine or fentanyl as an adjuvant to epidural bupivacaine for prevention of stump and phantom pain in adult patients undergoing above-knee or below-knee amputation: a randomized prospective trial. Research and Opinion in Anesthesia and Intensive Care. 2019;6(3):371.

3. Kuffler DP. Coping with phantom limb pain. Molecular neurobiology. 2018;55(1):70- 84.

4. Padovani MT, Martins MRI, Venâncio A, Forni JEN. Anxiety, depression and quality of life in individuals with phantom limb pain. Acta ortopedica brasileira. 2015;23(2):107-10.

5. Collins KL, Russell HG, Schumacher PJ, Robinson-Freeman KE, O’Conor EC, Gibney KD, et al. A review of current theories and treatments for phantom limb pain. The Journal of clinical investigation. 2018;128(6):2168-76.

6. Yildirim M, Sen S. Mirror Therapy in the Management of Phantom Limb Pain. AJN The American Journal of Nursing. 2020;120(3):41-6.

7. Nikolajsen L, Christensen KF. Phantom limb pain. Nerves and Nerve Injuries: Elsevier; 2015. p. 23-34.

8. Tureanu LM, Stojiljkovic L. Phantom Limb Pain. Chronic Pain Management in General and Hospital Practice: Springer; 2020. p. 261-77.

9. Münger M, Pinto CB, Pacheco‐Barrios K, Duarte D, Enes Gunduz M, Simis M, et al. Protective and Risk Factors for Phantom Limb Pain and Residual Limb Pain Severity. Pain Practice. 2020.

10. Suckow BD, Goodney PP, Nolan BW, Veeraswamy RK, Gallagher P, Cronenwett JL, et al. Domains that determine quality of life in vascular amputees. Annals of vascular surgery. 2015;29(4):722-30.

11. Ramadugu S, Nagabushnam SC, Katuwal N, Chatterjee K. Intervention for phantom limb pain: A randomized single crossover study of mirror therapy. Indian Journal of Psychiatry. 2017;59(4):457.

12. Tilak M, Isaac SA, Fletcher J, Vasanthan LT, Subbaiah RS, Babu A, et al. Mirror therapy and transcutaneous electrical nerve stimulation for management of phantom limb pain in amputees—a single blinded randomized controlled trial. Physiotherapy research international. 2016;21(2):109-15

13. Pereira ÂM, Ramos A, Rafaela A, João M, Arrifes V. Mobility in patients with lower limb amputation after prosthesis. Annals of Medicine. 2019;51(sup1):212-.

14. Wittkopf PG, Johnson MI. Mirror therapy: A potential intervention for pain management. Revista da Associação Médica Brasileira. 2017;63(11):1000-5.

15. Ambron E, Miller A, Kuchenbecker KJ, Buxbaum LJ, Coslett H. Immersive low-cost virtual reality treatment for phantom limb pain: Evidence from two cases. Frontiers in neurology. 2018;9:67.

16. Raffin E, Mattout J, Reilly KT, Giraux P. Disentangling motor execution from motor imagery with the phantom limb. Brain. 2012;135(2):582-95.

17. Aternali A, Katz J. Recent advances in understanding and managing phantom limb pain. F1000Research. 2019;8

18. Ülger Ö, Topuz S, Bayramlar K, Şener G, Erbahceci F. Effectiveness of phantom exercises for phantom limb pain: a pilot study. Journal of rehabilitation medicine. 2009;41(7):582-4.

**Research protocol: part 2**

**1. Budget**

No budget as such, research bears expenses, no financial support

**2. Other support for the project**

None.

**3. Curriculum Vitae of investigators**

Please find uploaded CV.

**4. Financing and insurance**

No funding sources.
